# Readability of Information on Smartphone Apps for Total Hip Replacement and Total Knee Replacement Surgery Patients

**DOI:** 10.1177/2374373519844266

**Published:** 2019-04-30

**Authors:** Shayan Bahadori, Thomas W Wainwright, Osman H Ahmed

**Affiliations:** 1 Executive Business Centre, Orthopaedic Research Institute, Bournemouth University, Bournemouth, United Kingdom; 2 Faculty of Health and Social Sciences, Bournemouth University, Bournemouth, United Kingdom; 3 The FA Centre for Disability Football Research, St Georges Park, Burton upon Trent, United Kingdom

**Keywords:** smartphone, apps, patient experience, total hip replacement, total knee replacement

## Abstract

**Background:**

Readability is a vital component of health information and providing this material at an appropriate literacy level may positively influence patient experience.

**Objective:**

To assess the readability of the information provided within total hip replacement and total knee replacement apps to understand more about the impact this could have on patients.

**Method:**

A systematic search was conducted across the 5 most popular smartphone app stores: iTunes, Google Play, Windows Mobile, Blackberry App World, and Nokia Ovi. Apps were identified for screening if they: targeted total hip replacement or total knee replacement patients; were free of charge; and were in English. App readability assessment was conducted independently by 3 reviewers using the Gunning Fog Index, the Flesch Reading Ease Score, and the Flesch-Kincaid Grade Level.

**Results:**

Fifteen apps met the inclusion criteria. Only one app was found “easy to read” (My THR).

**Conclusion:**

Findings suggest that the overall readability of information provided is written at a level which is difficult for patients to comprehend. App developers should engage patients in the design process of their apps, in order to enhance patient experience and for the potential impact of these innovative health technologies to be truly realized.

## Introduction

Total hip replacement (THR) and total knee replacement (TKR) surgeries are 2 of the most commonly undertaken procedures worldwide. However, the experience of the patient undergoing rehabilitation following THR and TKR can be highly variable (1). This may be due to the fact that written materials and patient information sheets have been found to vary in quality (2) and readability (3). Meanwhile, technical advances (in particular the widespread use of smartphones) have indicated that smartphone apps may have potential in enhancing rehabilitation and encouraging self-management following THR/TKR.

A recent systematic review examined the quality of smartphone apps targeted toward THR and TKR patients (4). It found that despite a wide range of apps currently available to THR and TKR patients, there is significant variability in their aesthetics, functionality, engagement, and quality of information (4). In addition to these aspects, readability is a vital component of health information and providing this material at an appropriate literacy level may positively influence patient experience. Existing readability scales such as the Gunning Fog Index (GFI), the Flesch Reading Ease Score (FRES), and the Flesch-Kincaid Grade Level (FKGL) have traditionally been used for assessing the formal education a person needs to understand the text on the first reading (5,6), and more recently health-care websites (5,7,8). To date however, there has been no evaluation of the readability of information on smartphone apps and how this might impact upon patients. This study aims to build on existing studies related to THR and TKR smartphone apps and to assess the readability of the information present on such apps in order to understand more about the impact this could have on patients.

## Method

The search strategy implemented in the study of Bahadori et al (4) was used to retrieve apps from the following sources: Android Google Play, Apple iTunes, BlackBerry World, Windows App Store, and Nokia Ovi Suite. These searches identified 2613 potentially relevant apps, of which 15 apps were include for analysis following the application of inclusion and exclusion criteria (4). Apps were excluded from evaluation if their primary focus was professional practice (ie, apps targeted toward surgeons/clinicians rather than patients); they were not available in the United Kingdom; required purchasing/special login access; outlined general physiotherapy exercises only; were a game (rather than an information app); were not in English; were an advertisement for a company; were solely journal or conference related; or were not related to THR or TKR.

The GFI, FRES, and FKGL are designed to indicate how difficult a passage in English is to understand (6,7). The GFI estimates the number of years of formal education required to understand the text on first reading. GFI scores range from 0 to 19+ and represent the reading level of the document. Scores of 0 to 6 correlate with low-literacy resources, 7 to 8 with resources comprehendible by junior high school students, 9 to 12 by high school students, 13 to 16 by college students, 17 to 18 by graduates, and 19 + by those with higher professional qualifications (9). An online tool was utilized to calculate the GFI for each app (10).

The FRES uses the length of sentences and the number of polysyllabic words to determine the overall FRES, while the FKGL utilizes the mean sentence and word length to calculate the complexity of the reading level (7). The FRES scores range from 0 to100, and a higher score is indicative of text that is easy to read (5). The FKGL scores range from 1 to 12 (corresponding to US educational school grades), with scores higher than 12 indicative of college level and domain-specific experts (5). The FRES is calculated using the formula 206.835 − (1.015 × average sentence lengths [ASLs]) − (84.6 × average number of syllables per word [ASW]). The FKGL is a modified version of the FRES scale and is calculated as (0.39 × ASL) + (11.8 × ASW) − 15.59. The inbuilt readability statistics feature of Microsoft Word 2007 was used to determine the FRES and FKGL for each app (7).

The lead author (S.B.) downloaded each of the apps and selected a body of text (approximately 150-250 words) from the main information page of each app (7). For each of the included apps the platform that they were available on was recorded, along with the clinical focus of the app (ie, THR, TKR, or both), provider (ie, governmental institution, nongovernmental institution [NGI]), and the characteristics of the app (ie, star rating, last update). The readability evaluation was carried out initially by the lead author (S.B.) and then repeated by one of the other members of the research team (T.W.W./O.H.A.), with verification occurring from cross-checking for consistency. Once all researchers had completed their assessment, results were pooled and where differences in the scoring existed, agreement was reached via consensus. This is in keeping with similar studies looking at readability of online health information (7).

## Results

The 15 apps included for analysis are outlined in Table 1 alongside their GFI, FRES, and FKGL data. Readability scores ranged from 6.4 to 10.9 (mean = 9.1, standard deviation [SD] = 1.4) for GFI, 46.4 to 89.4 (mean = 56.9, SD = 11.3) for FRES, and from 7 to 12.4 (mean = 9.7, SD = 1.3) for FKGL. Only one app (“My THR”) could be interpreted as “easy to read,” that is, it had a readability score of FKGL below 3.3, a GFI below sixth grade, and an FRES above 85.

**Table 1.** Basic Information of the Retrieved Apps and Their Readability Data.

Name of App	Platform	THR or TKR or Both	Star Rating	Last Updated	Provider^a^	FRES (0-100)	FKGL (**1**-12)	GFI
BeeWell Orthopaedic Hip	Apple iTunes	THR	NR	June 22, 2017	NGI	54.2	6.7	9.1
Hip Miss Samantha Z Troos	Apple iTunes	Both	NR	November 10, 2018	NGI	49.8	10.4	9.4
BeeWell Orthopaedic Knee	Apple iTunes	TKR	3.1	June 22, 2017	NGI	44.8	8.3	8.7
GreenCare Guide for Knee	Apple iTunes	TKR	NR	September 13, 2016	NGI	59.0	9.4	10.1
My Knee	Apple iTunes	TKR	NR	August 11, 2017	NGI	46.4	12.4	6.8
My Knee Guide	Apple iTunes	TKR	4.6	June 07, 2016	NGI	54.0	9.2	9.9
My THR	Google Play	THR	5.0	March 15, 2017	NGI	89.4	3.3	5.8
CommonSurgeries	Google Play	Both	3.9	March 05, 2015	NGI	48.9	10.2	7.0
Pocket Physio	Google Play	Both	4.3	April 06, 2016	NGI	54.7	6.0	9.3
Knee Pain Relieving	Google Play	TKR	4.4	December 31, 2016	NGI	74.6	5.5	10.9
Knee Pain Protocol	Google Play	TKR	4.6	December 02, 2018	NGI	63.5	7.0	10.6
Sport Injury Clinic	Google Play	Both	4.2	April 20, 2012	NGI	49.9	9.3	9.2
Ortho	Google Play	TKR	3.7	June 03, 2017	NGI	55.3	6.9	9.6
Know About Surgery treatment	Google Play	Both	NR	May 30, 2017	NGI	60.3	7.3	10.8
Healthy Knee	Blackberry	TKR	NR	August 05, 2015	NGI	49.3	8.9	8.6
Mean						56.9	9.7	9.1
Standard deviation						11.3	1.3	1.4

Abbreviations: FKGL, Flesch-Kincaid Grade Level; FRES, Flesch Reading Ease Score; GFI, Gunning Fog Index; NGI, nongovernmental institution; NR, none reported; THR, total hip replacement; TKR, total knee replacement.

^a^Type of provider is determined by answering “Credibility” questions 18 of MARS (11).

## Discussion

To the best of our knowledge, this is the first study to assess the readability of health information on smartphone apps. This is of importance because providing educational materials at an appropriate level of readability may help to enhance the patient experience.

An earlier evaluation of smartphone apps for THR and TKR patients suggested that despite a wide range of apps being available, there is significant variability in their quality (4). The findings from our study also suggest that the information on THR- and TKR-related smartphone apps is not written at a level which is easily comprehendible by the general public.

**Figure 1.**
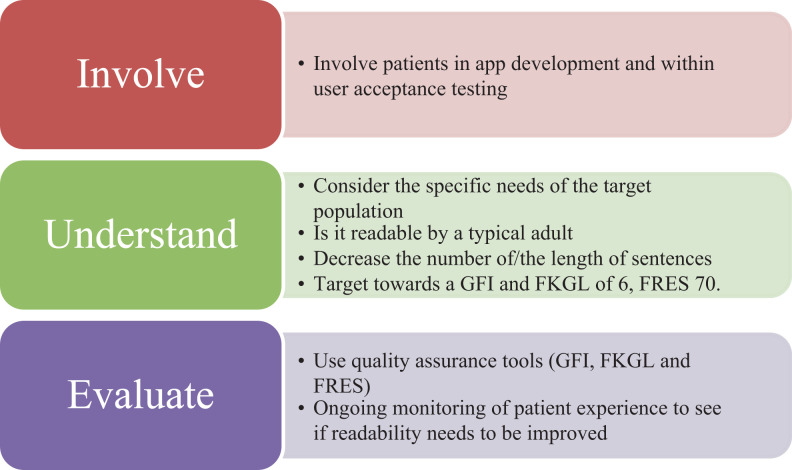
Recommendations for enhancing the readability of health-related apps.

This is of concern, given that differences in patient health literacy have been associated with differences in health outcomes (6,12,13). Previous work from the United States has shown that a significant proportion of the general public struggle with comprehending health-care information in the printed format (14), with the recommendation made that health information should be targeted toward an FKGL of around 6 (15). Furthermore, the available health information should be at a GFI level which is easy to understand by the general public and is not lost behind medical vocabulary (5). Practically, there are several steps that can be taken to improve the readability of health information within a smartphone app. Several of our key recommendations for ensuring readability of future THR and TKR apps (and other health-care-related apps) are highlighted in Figure 1.

In addition, all of the apps identified in this study were developed by NGIs, and as such no appraisal can be made regarding the association between the level of readability within apps and the type of provider. Inviting patients to participate in the evaluation and testing of health apps is an important stage in ensuring that readability requirements are met, and that the patient is at the center of this process.

## Conclusion

This study used the FRES and FKGL tools to evaluate the information on smartphone apps for THR and TKR patients. Findings suggest that the overall readability of information provided is written at a level which is difficult to read level for patients. App developers should engage patients in the design process of their apps, in order to enhance patient experience and for the impact of these innovative health technologies to be truly realized.
